# Primary Open Latarjet Procedure Results in Functional Differences but No Structural Changes in Subscapularis Muscle Quality vs the Healthy Contralateral Shoulder at Long-term Follow-up

**DOI:** 10.1177/03635465221079858

**Published:** 2022-03-22

**Authors:** Lukas Ernstbrunner, Manuel Waltenspül, Cyrill Suter, Rany El-Nashar, Johannes Scherr, Karl Wieser

**Affiliations:** † Department of Orthopedics, Balgrist University Hospital, University of Zurich, Zurich, Switzerland; ‡ Department of Orthopaedic Surgery, Royal Melbourne Hospital, Parkville, Australia; § Department of Biomedical Engineering, University of Melbourne, Parkville, Australia; ‖ Melbourne Orthopaedic Group, Windsor, Australia; Investigation performed at the Balgrist University Hospital, Zurich, Switzerland

**Keywords:** open Latarjet, subscapularis, internal rotation, external rotation, strength, isokinetic testing, 3D muscle volume, fat fraction

## Abstract

**Background:**

There are concerns that the Latarjet procedure results in loss of glenohumeral rotation and strength and in subscapularis dysfunction. The long-term effects of this procedure on subscapularis quality, glenohumeral rotation, and strength are unknown.

**Purpose/Hypothesis:**

To analyze the long-term effect of the primary open Latarjet procedure using a muscle-splitting approach on internal and external rotation and strength, as well as subscapularis muscle quality as compared with the healthy contralateral side. We hypothesized that the primary open Latarjet procedure is associated with a reduction of long-term shoulder strength and function and decreased subscapularis quality.

**Study Design:**

Case series; Level of evidence, 4.

**Methods:**

A total of 42 patients who underwent a primary open Latarjet procedure for recurrent anterior shoulder instability at a mean age of 26 years (range, 18-36) were reviewed after a mean follow-up of 8.4 years (range, 5-12). The subscapularis muscle volume and fat fraction of both shoulders were assessed. Bilateral active internal rotation (IR) and external rotation (ER), as well as IR and ER strength, were assessed by isokinetic testing (concentric, eccentric, and fatigability).

**Results:**

Active IR (0.6-point difference, *P* < .001) and ER (4° difference, *P* = .010) were significantly greater in healthy contralateral shoulders. The IR strength of the operated shoulder was significantly less than that of the healthy shoulder in concentric and eccentric testing (range of deficit, 4%-6%; *P* < .05). Also, the ER strength of the operated shoulder was significantly less than that of the healthy shoulder in concentric testing (11% deficit, *P* < .05). Subscapularis muscle volume was significantly greater in the operated shoulder (4% difference, *P* = .022), and there was no significant difference in fat fraction (*P* = .114).

**Conclusions:**

The primary open Latarjet procedure was associated with significantly decreased active IR and ER and strength when compared with the healthy contralateral shoulder. The clinical influence of these findings is yet to be defined. There was no increased subscapularis muscle fatty degeneration but a minimal hypertrophy on the operated side at long-term follow-up.

The Latarjet procedure^21^ was first described in 1954 and has undergone several modifications over time. The Latarjet procedure for recurrent anterior shoulder instability is very reliable, with reported long-term recurrence rates usually <10%.^1,9,12,14,18^ The stabilizing mechanisms of this procedure rely on the bone-block effect (ie, increasing glenoid surface area) as well as the sling effect (ie, dynamic tensioning of the lower part of subscapularis in abduction).^36^ A split in the subscapularis muscle is required to transfer the coracoid bone block through the muscle and fix it to the anteroinferior glenoid neck.^34^ The muscle split and the unphysiological tensioning of the subscapularis by the transferred conjoint tendon are matters of concern regarding the potential loss of glenohumeral rotation and strength.

With the technical advancement in shoulder stabilization procedures from a subscapularis takedown to a tendon-sparing and muscle-splitting approach, subscapularis function after the Latarjet procedure became more predictable with better range of motion (ROM), increased muscle power, and less fatty infiltration.^24^ Yet, the Latarjet procedure using a muscle-splitting approach is associated with loss of external rotation (ER)^14^ up to 19° and minimal loss of internal rotation (IR).^26^ Furthermore, short-term results focusing on subscapularis function after the Latarjet procedure revealed internal and external strength deficits and reduced endurance but no atrophy or fatty infiltration of the subscapularis muscle.^2^ The long-term effect of this procedure on shoulder function and strength as well as subscapularis quality is unclear.

It was therefore the question of this study whether the primary open Latarjet procedure using a muscle-splitting approach has an effect on long-term IR and ER, strength, and subscapularis muscle quality. We hypothesized that the primary open Latarjet procedure is associated with long-term reduction of IR and ER and strength, as well as subscapularis muscle atrophy and increased fatty infiltration, as compared with the healthy contralateral side.

## Methods

### Ethical Approval

This retrospective case-control study was approved by the Cantonal Ethics Committee (No. 2018-01929). All patients gave consent for the purpose of this study.

### Patients

Between January 2008 and December 2013, 105 consecutive patients aged 18 to 40 years were treated with an open Latarjet procedure for recurrent anterior shoulder instability at our institution. Patients were excluded from this study based on the following criteria: previous surgery on the affected side, revision of the Latarjet procedure, bilateral shoulder stabilization, contralateral shoulder instability or surgery, multidirectional instability, medical conditions affecting shoulder function (eg, rheumatoid arthritis), and incomplete imaging. Additionally, because of the pandemic, patients living abroad were not invited to the study, leaving 50 eligible patients.

**Figure 1.**
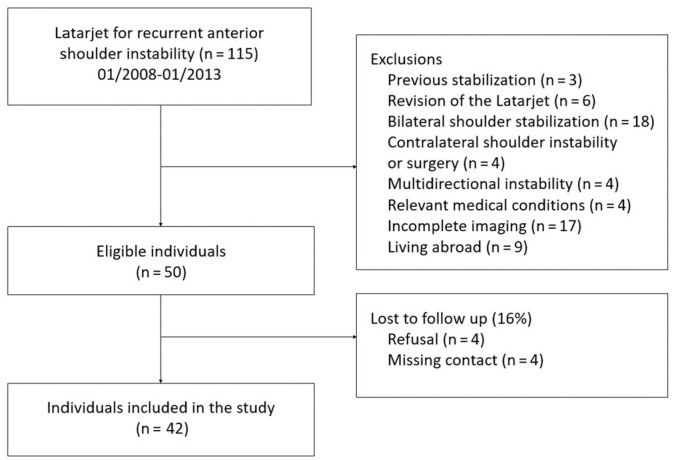
Flowchart of exclusion and lost to follow-up of study participants.

At final follow-up, 4 of these 50 could not be traced, and another 4 refused to participate but reported no complaints at the time of the telephone interview (Figure 1).

The reviewed cohort thus consisted of 42 patients (38 male, 4 female) and 84 shoulders with a mean age of 26 years (range, 18-36) at the time of the primary open Latarjet procedure. Patients were interviewed and examined at a mean follow-up of 8.4 years (range, 5-12). The decision for a primary open Latarjet procedure^30^ was based on preoperative glenoid bone loss >15% or if the patients had high physical activity levels or were heavy laborers.

### Surgical Technique

The surgical technique for the Latarjet procedure was performed according to the Walch refinement of the original technique described by Latarjet.^21,34^ Through a deltopectoral approach, the coracoid was osteotomized at its base using bent chisels. The glenoid neck was exposed through a horizontal split of the subscapularis muscle slightly below its midlevel while the arm was held in adduction and ER to tension the muscle. The harvested coracoid was positioned flush with the glenoid plane at the 2- to 5-o’clock position in a right shoulder and fixed with two 4.5-mm AO malleolar screws (Synthes) with the arm in a slightly abducted and neutral rotation position. The coracoacromial ligament was sutured to the most medial aspect of the incised capsule. The rotator cuff was inspected intraoperatively: the supraspinatus and infraspinatus tendons were inspected in combined IR, abduction, and extension and the subscapularis tendon in ER. No obvious pathology was detected.

The shoulder was immobilized for 4 weeks in a sling, and biceps activation and combined abduction and ER were restricted for 6 weeks. After 4 months, patients were allowed to return to sports.

### Isokinetic Testing

**Figure 2.**
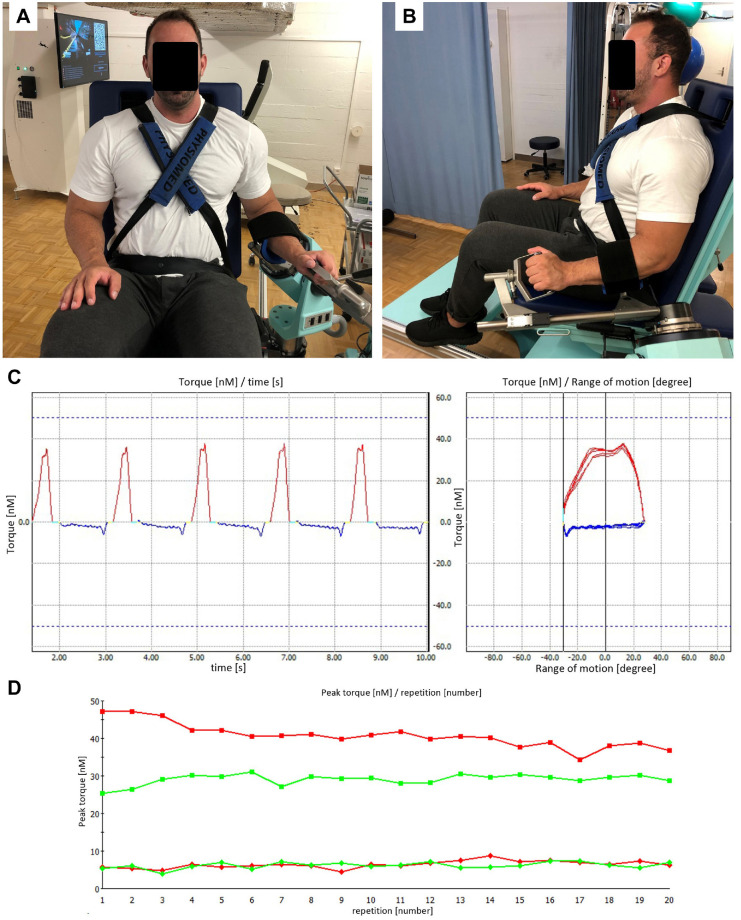
(A, B) Isokinetic testing of the left shoulder in 45° of abduction in the scapular plane and the elbow placed in neutral position and flexed at 90°. (C) Sequence in concentric mode with 5 repetitions at 240 deg/s indicating the torque (N·m) of each repetition in relation to time (left figure) and arc of motion (right figure). The red line indicates the torque of internal rotation, and the blue line indicates recovery during external rotation. (D) Peak torque per repetition during fatigue testing with 20 repetitions at 180 deg/s in concentric mode. The green and red lines indicate the internal rotation of the affected and healthy contralateral shoulders, respectively.

Isokinetic evaluation of both shoulders was performed using the Con-Trex MJ dynamometer (CMV AG). The patient was seated with the shoulder in 45° of abduction in the plane of the scapula according to the recommended testing position for IR and ER of the shoulder.^6,7^ The elbow was placed in neutral position and flexed at 90°. Each measurement starts from the “0° position,” and the ROM for each cycle was 60° and 30° for IR and ER, respectively. The patient performed a series of 5 tests with 1-minute recovery after each set. The following program was performed for each shoulder: in concentric mode, 5 repetitions at 240 deg/s and 180 deg/s and 3 repetitions at 60 deg/s; in eccentric mode, 5 repetitions at 60 deg/s; and finally, a fatigue test with 20 repetitions at 180 deg/s in concentric mode. At each angular velocity, dynamic strength of the IR and ER was evaluated using the measurement of the mean peak torque normalized to the patient’s body weight (N·m/kg). To assess the agonist-antagonist force-couple balance, the ER/IR mean peak torque ratio at each angular velocity (%) was calculated.^2^ The parameters were compared with the healthy contralateral shoulder (Figure 2).

### Assessment of Shoulder Function

Patients were examined to determine shoulder function as compared with the healthy contralateral side. Assessment of shoulder function included measurement of active IR described in Constant score (CS) points, ER with the arm at the side, absolute CS,^3^ the Western Ontario Shoulder Instability Index (WOSI) score,^19^ and the Subjective Shoulder Value (SSV).^15^ The minimum clinically important difference (MCID) for the CS was defined as 10 points,^22^ for the WOSI score as 220 points and 10%,^17^ and for the SSV as 12%.^8^

### Quantification of Subscapularis Muscle Volume and Fat Fraction

**Figure 3.**
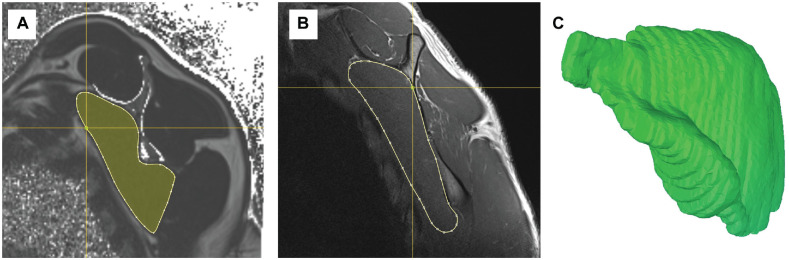
(A) Measurement of muscle fatty infiltration on sagittal magnetic resonance imaging 6-point Dixon sequences. (B) Margins of the subscapularis muscle in sectional planes (segments) of the whole cross section were manually marked on sagittal projections for assessment of muscle volume in MeVisLab. (C) The resulting 3-dimensional model presented in the Computer Assisted Surgery Planning Application (Balgrist CARD AG).

Bilateral magnetic resonance imaging (MRI) of the shoulders was obtained for volumetric and fat fraction analysis. All MRI scans were acquired at 3 T (Siemens MAGNETOM Prisma) using a dedicated 16-channel shoulder coil with sectional planes 3 mm thick. The MRI protocol was tailored for metal artifact reduction and included the following sequences: axial and paracoronal proton density Dixon, paracoronal and parasagittal STIR (short tau inversion recovery) with high bandwidth, and parasagittal T1 with high bandwidth. The Dixon sequences were used to generate fat signal fraction maps for subsequent intramuscular fat quantification.^5,16,35^ MRI scans were further used to measure the volume, length, and smallest enveloping box of the subscapularis muscle. The out-of-phase Dixon sequences were imported into MeVisLab (MeVis Medical Solutions AG), and sectional planes (segments) of the cross section of the supraspinatus muscle were manually outlined in sagittal projections with the freehand module of MeVisLab (Figure 3). The resulting segmented voxel data were converted into a 3-dimensional (3D) model with the generated WEM module to quantify muscle volume. In 1 patient, MRI had to be aborted because of claustrophobia, and the patient was excluded from final MRI analysis.

All MRI scans were assessed by 1 reader (C.S.), who was trained in 3D measurement in MeVisLab and fat fraction quantification. In the event of uncertainty, images were reassessed to achieve consensus with the first author (L.E.).

### Statistical Analysis

Based on a similar study on short-term results of IR strength after the open Latarjet procedure,^2^ an a priori power analysis revealed that for a significance level of .05 (type I error), a sample size of 42 patients was sufficient to provide a desired power of 80% to find a significant difference in IR strength in concentric and eccentric modes as compared with the healthy contralateral shoulder. All data were assessed for normality using the Shapiro-Wilk test. Comparison of the operated shoulder with the healthy contralateral shoulder was conducted using the paired *t* test (normal distribution) or the Wilcoxon signed-rank test (nonnormal distribution). The chi-square test or Fisher exact test (if n < 5) was used for categorical variables. Multivariate regression analyses were conducted to determine potential confounders of isokinetic testing. Significance level was set at *P* < .05.

## Results

The dominant shoulder was affected in 26 cases (62%) and the mean age at first-time dislocation was 20.4 years (range, 10-32). The preoperative amount of glenoid bone loss in the en face view was assessed by the Pico method on computed tomography scans13,23 and averaged 9% (range, 2%-32%). Three shoulders had preoperative mild glenohumeral osteoarthritis (OA; grade 1) according to Samilson and Prieto.1,27 Patients reported high preoperative activity levels, with 14 (33%) practicing overhead sports, 10 (24%) water sports, 7 (17%) contact sports, 5 (12%) various sports/activities, 4 (10%) board sports, and 2 (5%) heavy laborers.

### Isokinetic Testing

There was significantly lower IR strength (*P* < .05), except for the concentric test at 240 deg/s (*P* = .077), in the Latarjet shoulders as compared with the healthy contralateral shoulders, with a mean deficit ranging between 4% and 6%. There was also significantly lower ER strength (*P* < .05), except for the eccentric test at 60 deg/s (*P* = .326), in the Latarjet shoulders vs the healthy shoulders, with a mean deficit ranging between 4% and 11%. The Latarjet shoulders showed significantly less IR (*P* = .028) and ER (*P* = .002) endurance than the healthy shoulders (*P* < .05). The mean ER/IR ratio for the concentric test at 180 deg/s was significantly different between the Latarjet shoulders and the healthy shoulders (69% vs 74%, *P* = .039), indicating more balanced shoulders in the healthy group. Further details are listed in Table 1.

**Table 1:** Isokinetic Results of Internal and External Rotators^a^

	Mean (± SD) Peak Torque ^b^			
Testing condition^b^	Healthy (n = 42)	Latarjet (n = 42)	Deficit, %	*P* Value
IR				
Concentric test^c^				
240 deg/s	0.57 ± 0.12	0.55 ± 0.13	4	.077
180 deg/s	0.60 ± 0.13	0.57 ± 0.13	5	.**035**
60 deg/s	0.64 ± 0.14	0.60 ± 0.14	6	.**007**
Eccentric test: 60 deg/s	0.49 ± 0.12	0.47 ± 0.88	4	.**033**
Fatigue test: 180 deg/s^d^	0.48 ± 0.13	0.45 ± 0.12	6	.**028**
ER				
Concentric test^c^				
240 deg/s	0.44 ± 0.93	0.39 ± 0.87	11	**<.001**
180 deg/s	0.44 ± 0.09	0.39 ± 0.09	11	**<.001**
60 deg/s	0.44 ± 0.09	0.39 ± 0.09	11	**<.001**
Eccentric test: 60 deg/s	0.50 ± 0.14	0.48 ± 0.17	4	.326
Fatigue test: 180 deg/s^d^	0.38 ± 0.08	0.36 ± 0.07	5	.**002**
ER/IR ratio				
Concentric				
240 deg/s	0.79 ± 0.16	0.74 ± 0.15		.096
180 deg/s	0.74 ± 0.12	0.69 ± 0.15		.**039**
60 deg/s	0.70 ± 0.13	0.66 ± 0.15		.156
Eccentric : 60 deg/s	1.01 ± 0.26	1.03 ± 0.45		.087

a Bold indicates *P* < .05. ER, external rotation; IR, internal rotation.

b Mean peak torque was normalized to the patient’s body weight (N·m/kg).

c Two patients aborted the concentric testing and were excluded from analysis.

d Two patients aborted the fatigue testing and were excluded from analysis.

Multivariate analysis revealed that sex was the only significant confounding factor of isokinetic testing (*P* < .05). Factors such as dominance, age, and body mass index had no significant influence on isokinetic measurements (*P* > .05).

### Shoulder Function

Active IR (*P* < .001) and ER (*P* = .010), CS (*P* < .001), and SSV (*P* < .001) were significantly higher, and the WOSI score (*P* < .001) significantly lower, in the unaffected shoulders. Detailed information about shoulder function is provided in Table 2.

**Table 2:** Postoperative Clinical Results vs the Healthy Contralateral Side^a^

	Healthy (n = 42)	Latarjet (n = 42)	Deficit	*P* Value
External rotation, deg	63 ± 17	59 ± 19	4	.**010**
Constant score				
Internal rotation (CS points)	9.6 ± 0.8	9.0 ± 1	0.6	.**001**
Absolute	91 ± 5	87 ± 7	4	**<.001**
WOSI				
Points	135 ± 171	375 ± 351	240	**<.001**
%	6 ± 8	18 ± 17	12	**<.001**
Subjective Shoulder Value, %	93 ± 10	84 ± 17	9	**<.001**

a Bold indicates *P* < .05. CS, Constant score; WOSI, Western Ontario Shoulder Instability Index.

### Subscapularis Muscle Volume and Fat Fraction

Assessment of subscapularis quality revealed significantly larger muscle volume in the Latarjet shoulders as compared with the healthy shoulders (177 vs 169 cm^3^, 4% difference, *P* = .022). There was no significant difference in subscapularis fat fraction between the Latarjet shoulders and the healthy shoulders (3.8% vs 4.0%, *P* = .114).

## Discussion

The main finding of this study about the long-term functional and structural effect of the primary open Latarjet procedure using a tendon-sparing and muscle-splitting approach is that it does lead to a significant loss in IR strength by about 5% and a significant and more pronounced loss in concentric ER strength of 11% as compared with the healthy contralateral shoulder. It is also associated with significantly more IR and ER fatigability and significantly less active glenohumeral rotation. Despite the significant differences in function, the clinical implications of the observed mild loss in IR and ER, strength, and endurance are yet to be defined. Furthermore, the procedure does not lead to subscapularis atrophy or increased fatty infiltration as compared with the healthy contralateral shoulder.

With the progression from a subscapularis tenotomy toward a tendon-sparing and muscle-splitting approach, devastating complications such as significant degeneration and subscapularis dysfunction became less frequent in open shoulder stabilization procedures. When compared with tenotomy, the tendon-sparing and muscle-splitting approach results in significantly greater amplitude of IR, increased muscle power, and less fatty infiltration.^24^

One reason for possible subscapularis dysfunction and fatty degeneration after subscapularis tenotomy is denervation during the surgical approach with release and mobilization of the musculotendinous unit, in particular the upper part of the subscapularis.^25,28,29^ Notwithstanding, the Latarjet procedure with a tendon-sparing approach still creates a permanent split in the subscapularis muscle. This split is caused by the transferred conjoint tendon, which tensions the inferior part of the subscapularis and thereby causes a sling effect, one of the main stabilizing mechanisms of this procedure.^36^ Besides this stabilizing effect, there are concerns that this permanent redirection of the muscle and tendon may jeopardize subscapularis structure and function.^31^ Although the consequence of this redirection may solely be altering the moment arm of the subscapularis, the integrity of the inferior half of the subscapularis is without any doubt one of the prerequisites of a stable and functioning glenohumeral joint.^10,11^

The Latarjet procedure was reported to be associated with varying loss of glenohumeral rotation strength. Dauty et al^4^ cited a deficit of 9% to 15% for IR when testing in the eccentric mode 3 months after surgery. Caubère et al^2^ showed that the open Latarjet procedure resulted in a strength deficit in IR and ER ranging from 13% to 20% 1 year after the procedure. They also found reduced endurance compared with the healthy shoulder. The current study confirms these findings of a slight but significant loss in IR strength, between 4% and 6%, and a more pronounced loss of ER strength, 11% in the concentric mode and 4% in the eccentric mode, at a long-term follow-up. In accordance with the short-term functional results of Caubère et al, the shoulders treated with the Latarjet procedure in our study revealed significantly more fatigability in IR and ER as compared with the healthy shoulders. Contrary to these short-term results, we found that in the fastest concentric testing mode (240 deg/s), the Latarjet shoulders were significantly less balanced between internal and external rotators than were the healthy shoulders the same testing mode.

The Latarjet procedure is associated with loss of active ROM, loss of active ER^14^ up to 19°, and minimal loss of active IR.^26^ In the current study, we included patients without any previous or revision surgery, with clear recurrent anterior instability before the Latarjet procedure, and without any contralateral shoulder pathology or multidirectional instability. Based on these strict inclusion and exclusion criteria, the loss in active IR and ER after a primary open Latarjet procedure as compared with the healthy shoulder at long-term follow-up was only 4° of ER and about half a CS point for IR, with a mean active IR in both shoulders above the T12 vertebra. The effect of reduced postoperative ROM on clinical outcome remains unclear. Lafosse and Boyle^20^ reported a return to the previous level of activity in 35 patients after the arthroscopic Latarjet procedure, despite a restricted ER of 18°. Similarly, in a recent study Sinha et al^32^ showed that a decrease in ER of 10° and IR of 6° after the open Latarjet procedure had a negligible effect on return to activity at short-term follow-up. In the current study, the Latarjet shoulders also had a significantly lower CS and SSV and a higher WOSI score than the healthy shoulders. The differences in CS and SSV did not reach the MCID of 10 points^22^ and 12%,^8^ respectively. However, the difference in WOSI score was above the MCID of 220 points and 10%.^17^

To answer the question of the effect of the primary open Latarjet procedure on subscapularis muscle quality, the current study used a detailed and previously published 3D muscle segmentation protocol for analysis of subscapularis quality.^35^ It showed that the primary open Latarjet procedure is not associated with atrophy or with fatty infiltration as compared with a completely healthy shoulder, which is in accordance with a previous short-term study.^2^ Interestingly, a slight but significant subscapularis hypertrophy on the operated side was observed. A reason for this might be a compensatory hypertrophy of the subscapularis attributed to a change in moment arm by the sling effect after the Latarjet procedure. However, hypertrophy of the subscapularis was also reported after open iliac bone grafting for recurrent anterior shoulder instability, with up to a quarter of subscapularis hypertrophy measured in the operated shoulder versus the contralateral side.^33^ Further biomechanical studies are needed to investigate if this hypertrophy can be related to a change in moment arm.

The following limitations have to be acknowledged. First, this was a retrospective comparative case-control study, with all its associated biases. Selection bias was minimized by reviewing all patients with an open Latarjet procedure between the age of 18 to 40 years and by using strict inclusion and exclusion criteria as outlined earlier. With a minimum follow-up of 5 years and a follow-up rate of 84%, the provided results are a robust basis for further studies. We did not perform preoperative and early postoperative baseline MRI and isokinetic testing, which would have been useful for evaluation of preoperative muscle status as well as recovery after the open Latarjet procedure. Furthermore, we had no control groups of patients after other surgical stabilization procedures, such as an arthroscopic Bankart repair or an iliac crest bone-block procedure. We are also not aware of such data in recent literature. It remains therefore unanswered whether other stabilization procedures have different effects on rotational strength and endurance. Another limitation is that we did not perform radiographs of the healthy shoulders to compare glenohumeral OA grades with the Latarjet shoulders. As all Latarjet shoulders had no or only mild radiographic signs of OA (ie, grade 1 or 2), the effect of arthritic changes on ROM should not be relevant for the interpretation of the results.

Despite these limitations, this is the first study reporting on long-term results of bilateral shoulder function, isokinetic testing, and 3D muscle segmentation analysis of a very strictly selected cohort of patients with a primary open Latarjet procedure for recurrent anterior shoulder instability and healthy contralateral shoulders. Overall, the observed loss in glenohumeral rotation and strength seems not to be of strong clinical relevance. When a patient is counseled about the Latarjet procedure, the provided information will help him or her understand what can be expected in terms of subscapularis quality, shoulder function, and strength 8 years after a primary open Latarjet procedure.

## Conclusion

The primary open Latarjet procedure is associated with significantly decreased active IR and ER and strength when compared with the healthy contralateral shoulder. The clinical influence of these findings is yet to be defined. There is no increased subscapularis muscle fatty degeneration but minimal hypertrophy on the operated side at long-term follow-up.
